# Severity-associated markers and assessment model for predicting the severity of COVID-19: a retrospective study in Hangzhou, China

**DOI:** 10.1186/s12879-021-06509-6

**Published:** 2021-08-09

**Authors:** Jianjiang Qi, Di He, Dagan Yang, Mengyan Wang, Wenjun Ma, Huaizhong Cui, Fei Ye, Fei Wang, Jinjian Xu, Zhijian Li, Chuntao Liu, Jing Wu, Kexin Qi, Rui Wu, Jinsong Huang, Shourong Liu, Yimin Zhu

**Affiliations:** 1 Hangzhou Xixi Hospital, Hangzhou, 310023 Zhejiang China; 2 Department of Epidemiology & Biostatistics, School of Public Health, Zhejiang University, Hangzhou, 310058 Zhejiang China; 3 The First Affiliated Hospital, School of Medicine, Zhejiang University, Hangzhou, 310003 Zhejiang China; 4 Department of Pathology, Zhejiang University School of Medicine, Hangzhou, 310058 Zhejiang China

**Keywords:** COVID-19, Severity, Assessment model, Prediction, Web-based assessment system

## Abstract

**Background:**

The severity of COVID-19 associates with the clinical decision making and the prognosis of COVID-19 patients, therefore, early identification of patients who are likely to develop severe or critical COVID-19 is critical in clinical practice. The aim of this study was to screen severity-associated markers and construct an assessment model for predicting the severity of COVID-19.

**Methods:**

172 confirmed COVID-19 patients were enrolled from two designated hospitals in Hangzhou, China. Ordinal logistic regression was used to screen severity-associated markers. Least Absolute Shrinkage and Selection Operator (LASSO) regression was performed for further feature selection. Assessment models were constructed using logistic regression, ridge regression, support vector machine and random forest. The area under the receiver operator characteristic curve (AUROC) was used to evaluate the performance of different models. Internal validation was performed by using bootstrap with 500 re-sampling in the training set, and external validation was performed in the validation set for the four models, respectively.

**Results:**

Age, comorbidity, fever, and 18 laboratory markers were associated with the severity of COVID-19 (all *P* values < 0.05). By LASSO regression, eight markers were included for the assessment model construction. The ridge regression model had the best performance with AUROCs of 0.930 (95% CI, 0.914–0.943) and 0.827 (95% CI, 0.716–0.921) in the internal and external validations, respectively. A risk score, established based on the ridge regression model, had good discrimination in all patients with an AUROC of 0.897 (95% CI 0.845–0.940), and a well-fitted calibration curve. Using the optimal cutoff value of 71, the sensitivity and specificity were 87.1% and 78.1%, respectively. A web-based assessment system was developed based on the risk score.

**Conclusions:**

Eight clinical markers of lactate dehydrogenase, C-reactive protein, albumin, comorbidity, electrolyte disturbance, coagulation function, eosinophil and lymphocyte counts were associated with the severity of COVID-19. An assessment model constructed with these eight markers would help the clinician to evaluate the likelihood of developing severity of COVID-19 at admission and early take measures on clinical treatment.

**Supplementary Information:**

The online version contains supplementary material available at 10.1186/s12879-021-06509-6.

## Background

Coronavirus disease 2019 (COVID-19) is caused by severe acute respiratory syndrome coronavirus 2 (SARS-CoV-2) and has spread worldwide [1]. On March 12, 2020, the World Health Organization (WHO) announced the disease to be pandemic. It has affected more than 200 countries with about 10,000,000 confirmed cases as of July 01, 2020 [2]. Therefore, the epidemic of COVID-19 has become a global public health crisis.

Different clinical patterns, such as mild, moderate, and severe to critical types, were observed in patients with COVID-19. Although most COVID-19 patients have mild or moderate symptoms and signs, the finding from China indicated that about 14% of patients were of the severe type and 5% were of the critical type [3]. Previous studies and clinical practice showed that the degree of severity was associated with the clinical treatment and prognosis of the disease [3–6]. The average overall case-fatality rate of confirmed COVID-19 patients was 2.3%, but that was up to 49.0% in critical patients [3]. Missed diagnoses will delay the appropriate clinical treatment and increase the possibility of poor prognosis. On the other hand, treatment for a severe or critical COVID-19 patient requires vast medical resources, and over misdiagnoses will overuse the medical resources and increase the medical burden. Therefore, early identification of patients who are likely to develop severe or critical COVID-19 is especially important for clinical practice and epidemic control. In clinical practice, the severity of COVID-19 is categorised into four levels as mild, moderate, severe, and critical types according to the Seventh Edition of the Guide to Diagnosis and Treatment of New Coronary Pneumonia [7]. This classification is preformed mainly based on the clinical symptoms, oxygen saturation (SaO2), and imaging evidence from computed tomography (CT). However, no evidence from laboratory markers has been included. Previous studies have found that lymphopenia, organ dysfunction, coagulopathy, and elevated D-dimer levels were associated with the severity [3–6, 8].

In this study, we aimed to screen severity-associated markers and construct an assessment model for predicting the severity of patients with COVID-19 based on the data from two hospitals in Hangzhou, Zhejiang province, China.

## Methods

### Study population

This study enrolled 172 confirmed COVID-19 patients from January 20, 2020 to April 1, 2020 in Hangzhou, Zhejiang Province, China. Among these patients, 104 from Hangzhou Xixi Hospital were used for screening the severity-associated markers and constructing the assessment model as a training set. Part of the 104 patients had been used in the previously published studies [9, 10]. On the other hand, 68 patients from the First Affiliated Hospital, School of Medicine, Zhejiang University (FAHZJU) were used to validate the model as a validation set. These patients were part of the sample which had been published previously [10]. COVID-19 was diagnosed according to the interim guidance from the WHO [11]. The severity of COVID-19 was categorised into four levels according to the Seventh Edition of the Guide to Diagnosis and Treatment of New Coronary Pneumonia [7]. The mild type was defined as patients with mild clinical symptoms and normal imaging on CT. The moderate type was defined as patients with fever, respiratory symptoms, or other symptoms, and altered imaging evidence with pneumonia. The severe type was defined as patients with at least one of the following symptoms: shortness of breath (breathing rate ≥ 30/min), SaO_2_ at rest ≤ 93%, partial pressure of oxygen in arterial blood (PaO_2_)/ inspired oxygen fraction (FiO_2_) ≤ 300 mmHg, or lung infiltrates > 50% within 24 to 48 h. The critical type was defined as patients with any of the following symptoms: respiratory failure requiring mechanical ventilation, shock, or a combination of other organ failures requiring ICU monitoring treatment.

This was a retrospective study and the protocol was approved by the Ethics Committee of Xixi Hospital and FAHZJU.

### Data collection

Data at admission, including demographic information, comorbidities, clinical symptoms and laboratory tests, were extracted from electronic medical records. Collected data were reviewed by a trained team of clinical physicians. Demographic information included age, sex and body mass index (BMI). Comorbidity was defined as having at least one of the following diseases: diabetes, hypertension, cardiovascular disease, severe congenital disease, cancer, and chronic diseases of the liver, kidney, or respiratory system. Clinical symptoms included fever, fatigue, cough, expectoration, shortness of breath, diarrhoea and myalgia. Laboratory markers of laboratory tests included the following eight categories: inflammation, electrolytes, nutritional metabolism, and liver, renal, cardiac, respiratory, coagulation functions.

### Statistical analysis

Continuous variables were presented as median (interquartile range [IQR]), and categorical variables were presented as numbers (percentage). Continuous laboratory markers were dichotomously categorised (normal versus abnormal) under the criteria of their clinical reference values. Severity-associated markers of COVID-19 were screened using the ordinal logistic regression.

To construct an assessment model, two criteria were set for selecting markers: P value < 0.05 in the ordinal logistic regression, and at least half of severe or critical patients had an abnormality in the marker. Least Absolute Shrinkage and Selection Operator (LASSO) regression was used for further feature selection. Optimal regularization parameter (λ) was estimated by fivefold cross-validation. To increase the stability of feature selection, we used bootstrap with 1000 resamples and built a LASSO regression model for each bootstrap set. The markers, which were present in more than half of all bootstrap sets, were included in the final model.

Assessment models were constructed using logistic regression, ridge regression, support vector machine, and random forest in the training set. The performance of different models was evaluated by the area under the receiver operator characteristic curve (AUROC). For the internal validation, we used bootstrap with 500 resamples to decrease the over-fitting. For the external validation, four models were assessed in the validation set, respectively. A risk score was established according to the result of the best model. The performance of the risk score in all patients was evaluated using AUROC and calibration curve. The optimal cutoff value was calculated with the maximal Youden index. A web-based assessment system was developed based on the risk score.

All statistical analyses were conducted using R software, version 3.6.2 (R Foundation for Statistical Computing). A two-sided P value < 0.05 was considered statistically significant.

## Results

### Basic characteristics of the study population

**Fig. 1:**
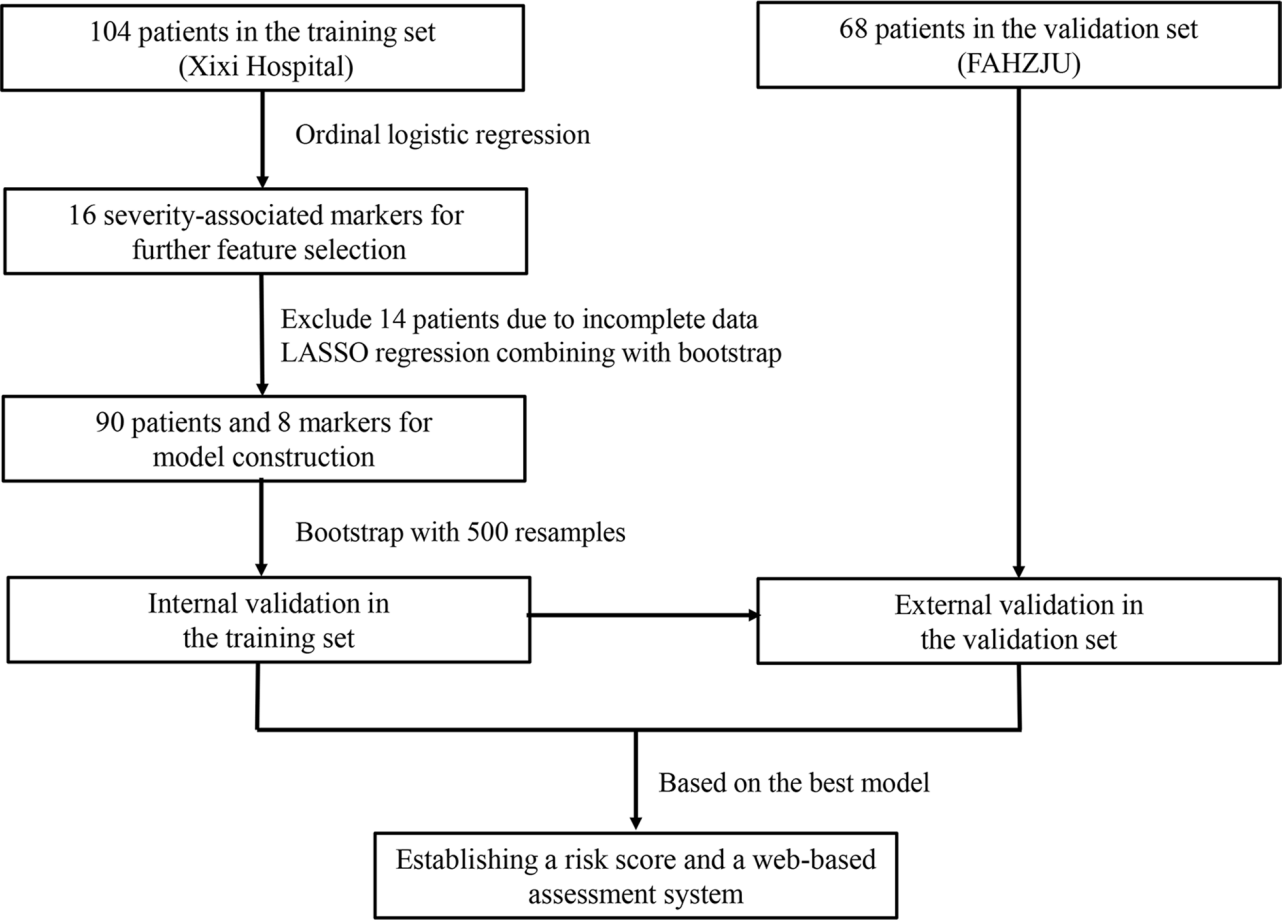
Flowchart of the study procedure

**Fig. 2:**
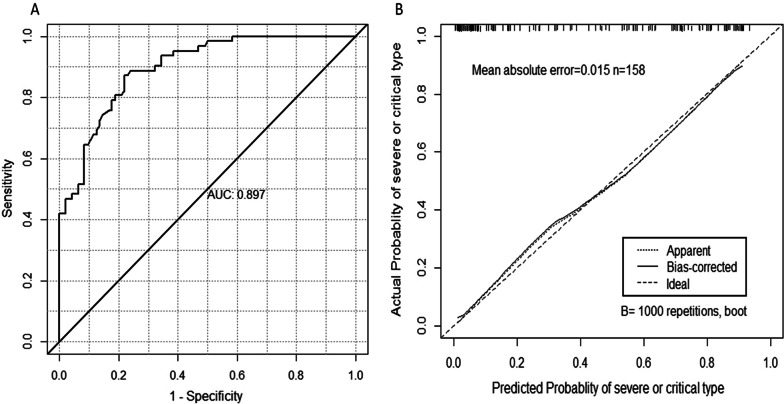
The performance assessment of the risk score in all patients. **A** The receiver operator characteristic curve of the risk score. **B** The calibration curve of the risk score with bootstrap

**Fig. 3:**
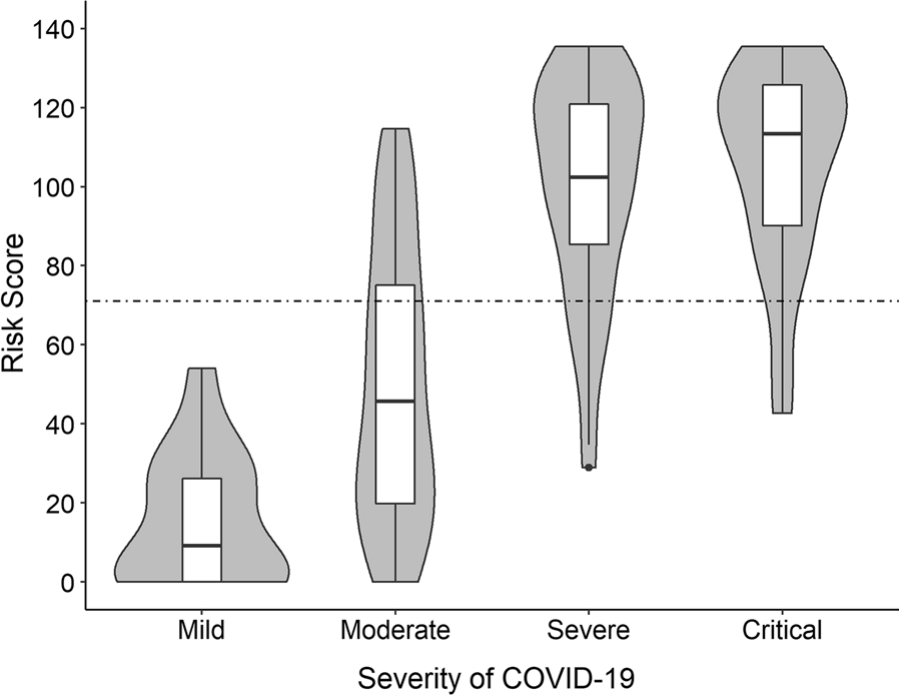
Violin plots of the risk scores in different degrees of COVID-19 severity. Width of the violin plots represented the frequency of the risk score in that level. Each box represented the lower quartile (25%), median (50%) and upper quartile (75%). The horizontal dashed line represented the cutoff value of the risk score (71)

**Fig. 4:**
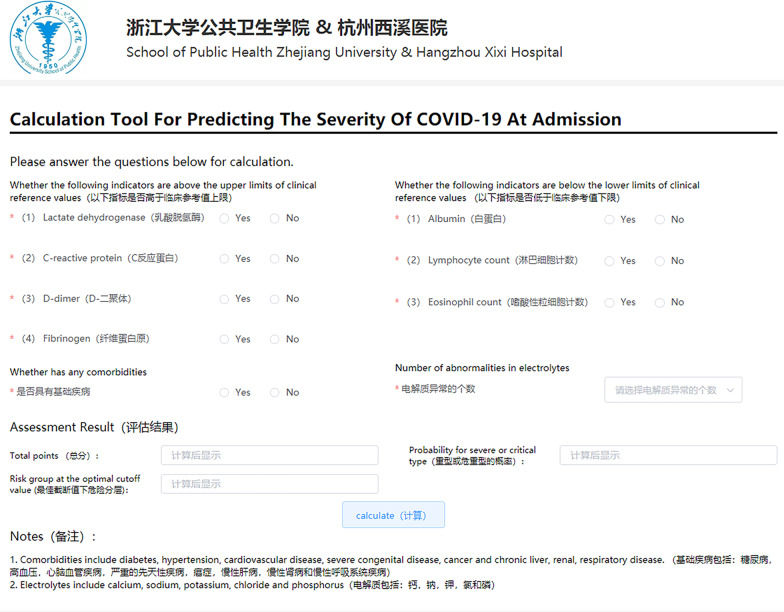
The online calculator for assessing the severity of COVID-19 at admission. Website: http://www.gtrsp.com:8011/

The flowchart of the study procedure is illustrated in Fig. 1. Basic characteristics of the COVID-19 patients are summarised in Table 1. The patients in the training set had a median age of 42.0 years (IQR: 33.0–56.5) and a median BMI of 22.5 kg/m^2^ (IQR: 20.3–25.0). Among them, 47(45.2%) patients were men, and 23 (22.1%) patients had at least one comorbidity. During hospitalisation, 21 (20.2%) patients were classified as mild type, 72 (69.2%) as moderate type, and 11 (10.6%) as severe type. In the validation set, the median age and BMI were 59.0 years (IQR: 48.0–66.0) and 24.7 kg/m^2^ (IQR: 22.1–27.0), respectively. 44 (64.7%) patients were men, and 56 (82.4%) patients had at least one comorbidity. During hospitalisation, 16 (23.5%) patients were classified as moderate type, 29 (42.7%) as severe type, and 23 (33.8%) as critical type. The most common clinical symptoms were fever and cough, followed by expectoration and shortness of breath in both the training and validation sets (Figs. 2, 3 and 4.

**Table 1:** Basic characteristics of the study population

	Training set (n = 104)	Validation set (n = 68)
Age (years), median (IQR)	42.0 (33.0–56.5)	59.0 (48.0–66.0)
BMI (kg/m^2^), median (IQR)	22.5 (20.3–25.0)	24.7 (22.1–27.0)
Sex, n (%)		
Male	47 (45.2)	44 (64.7)
Female	57 (54.8)	24 (35.3)
Comorbidity^a^, n (%)		
Yes	23 (22.1)	56 (82.4)
No	81 (77.9)	12 (17.6)
Degree of severity, n (%)		
Mild	21 (20.2)	0 (0)
Moderate	72 (69.2)	16 (23.5)
Severe	11 (10.6)	29 (42.7)
Critical	0 (0)	23 (33.8)
Clinical symptoms, n (%)		
Fever	69 (66.3)	58 (85.3)
Fatigue	6 (5.8)	13 (19.1)
Cough	46 (44.2)	42 (61.8)
Expectoration	9 (8.7)	28 (41.2)
Shortness of breath	8 (7.7)	19 (27.9)
Diarrhoea	2 (1.9)	7 (10.3)
Myalgia	2 (1.9)	18 (26.5)

^a^Comorbidity was defined as having at least one of the following diseases: diabetes, hypertension, cardiovascular disease, severe congenital disease, cancer and chronic liver, renal, respiratory disease

*IQR* Interquartile range; *BMI* Body mass index

### Severity-associated markers of COVID-19

Table 2 presents the associations of clinical characteristics with the severity of COVID-19 in the training set. For demographic characteristics and clinical symptoms, age, comorbidity, and fever were associated with the severity of COVID-19 (all P values < 0.05). For dichotomous laboratory markers, higher levels of C-reactive protein (CRP), lactate dehydrogenase (LDH), serum amyloid A, fibrinogen (FIB), D-dimer, adenosine deaminase, reduced haemoglobin, and lower levels of lymphocyte, eosinophil, platelet counts, calcium, phosphorus, albumin (ALB), albumin/globulin, prealbumin, total cholesterol, high density lipoprotein cholesterol, retinol binding protein, apolipoprotein A1, SaO_2_, PaO_2_/FiO_2_ increased the risk of elevated COVID-19 severity (all P values < 0.05). Detailed results of the associations of continuous laboratory markers data with COVID-19 severity are summarised in Additional file 1: Table S1.

**Table 2:** Associations of clinical characteristics with the severity of COVID-19 in the training set

	Mild^a^	Moderate^a^	Severe^a^	OR (95% CI)	*P* value
Number	21	72	11		
Demographic characteristics					
Age (years)	30.0 (14.5–40.5)	44.5 (34.3–57.0)	60.0 (45.0–72.0)	1.07 (1.04–1.11)	< 0.001
Female	8 (38.1)	44 (61.1)	5 (45.5)	1.52 (0.66–3.48)	0.326
BMI (kg/m^2^)	21.2 (18.1–25.4)	22.5 (20.4–25.1)	23.4 (20.4–24.0)	1.08 (0.96–1.22)	0.218
Comorbidity^b^	1 (4.8)	15 (20.8)	7 (63.6)	8.09 (2.46–26.55)	< 0.001
Clinical symptoms					
Fever	10 (47.6)	49 (68.1)	10 (90.9)	3.11(1.26–7.66)	0.014
Fatigue	0 (0)	5 (6.9)	1 (9.1)	2.89(0.49–16.95)	0.240
Cough	7 (33.3)	34 (47.2)	5 (45.5)	1.47(0.64–3.39)	0.367
Expectoration	2 (9.5)	5 (6.9)	2 (18.2)	1.51(0.34–6.65)	0.584
Shortness of breath	1 (4.8)	6 (8.3)	1 (9.1)	1.51(0.32–7.22)	0.604
Diarrhoea	1 (4.8)	1 (1.4)	0 (0)	0.23(0.01–3.72)	0.301
Myalgia	1 (4.8)	1 (1.4)	0 (0)	0.23(0.01–3.72)	0.301
Laboratory tests					
WBC count (< 3.5 × 10^9^/L)	1 (4.8)	5 (6.9)	2 (18.2)	2.66 (0.56–12.66)	0.220
Lymphocyte count (< 1.1 × 10^9^/L)	5 (23.8)	31 (43.1)	7 (63.6)	2.73 (1.11–6.68)	0.028
Eosinophil count (< 0.02 × 10^9^/L)	4 (19.0)	39 (54.2)	9 (81.8)	5.63 (2.08–15.18)	0.001
Platelet count (< 125 × 10^9^/L)	0 (0)	5 (6.9)	3 (27.3)	7.52 (1.62–34.98)	0.010
CRP (> 10 mg/L)	2 (9.5)	30 (41.7)	10 (90.9)	12.08(3.38–43.21)	< 0.001
LDH (> 250 U/L)	0 (0)	10 (14.1)	6 (54.5)	11.55 (3.14–42.52)	< 0.001
SAA (> 10 mg/L)	11 (52.4)	60 (83.3)	11 (100)	5.97 (2.13–16.67)	0.001
Calcium (< 2.2 mmol/L)	0 (0)	19 (27.5)	9 (81.8)	22.69 (4.77–108.01)	< 0.001
Potassium (< 3.5 mmol/L)	4 (19.0)	20 (27.8)	3 (27.3)	1.38 (0.53–3.56)	0.506
Phosphorus (< 0.81 mmol/L)	0 (0)	14 (20.3)	3 (27.3)	3.58 (1.10–11.6)	0.034
Chlorine (< 99 mmol/L)	0 (0)	9 (12.5)	2 (18.2)	3.36 (0.85–13.24)	0.083
Sodium (< 137 mmol/L)	6 (28.6)	25 (34.7)	7 (63.6)	2.17(0.89–5.28)	0.090
ALB (< 40 g/L)	3 (14.3)	30 (41.7)	11 (100)	12.12(3.41–43.11)	< 0.001
ALB/GLB (< 1.2)	0 (0)	18 (25.0)	7 (63.6)	10.34 (2.98–35.85)	< 0.001
PALB (< 180 mg/L for females and < 200 mg/L for males)	7 (35.0)	49 (74.2)	11 (100)	7.70 (2.74–21.68)	< 0.001
TG (> 1.7 mmol/L)	5 (23.8)	13 (18.1)	3 (27.3)	0.98 (0.35–2.73)	0.975
TC (< 3 mmol/L)	0 (0)	3 (4.2)	2 (18.2)	7.41 (1.16–47.21)	0.034
HDL-C (< 1.1 mmol/L for females and < 1.04 mmol/L for males)	4 (19.0)	32 (44.4)	8 (72.7)	4.06 (1.57–10.52)	0.004
RBP (< 23 mg/L)	5(25.0)	36(54.5)	8 (80.0)	4.20 (1.60–11.03)	0.004
Apo A1 (< 1 g/L)	10 (50.0)	43 (65.2)	10 (100)	3.20 (1.26–8.15)	0.015
GLB (> 40 g/L)	0 (0)	3 (4.2)	0 (0)	1.48 (0.12–17.76)	0.756
FIB (> 3.5 g/L)	4 (19.0)	30 (41.7)	9 (81.8)	5.00 (1.84–13.54)	0.002
D-dimer (> 0.55 mg/L)	2 (9.5)	12 (16.7)	6 (54.5)	4.91 (1.57–15.41)	0.006
SaO_2_ (≤ 93%)	0 (0)	4 (5.7)	3 (27.3)	8.33 (1.64–42.30)	0.011
PaO_2_/FiO_2_ (≤ 300 mmHg)	0 (0)	4 (5.7)	3 (27.3)	8.33 (1.64–42.30)	0.011
PaCO_2_ (< 35 mmHg)	1 (6.2)	6 (8.6)	3 (27.3)	3.43 (0.82–14.41)	0.093
ALT (> 40 U/L for females and > 50 U/L for males)	4 (19.0)	6 (8.3)	2 (18.2)	0.68 (0.19–2.41)	0.553
AST (> 35 U/L for females and > 40 U/L for males)	4 (19.0)	8 (11.1)	5 (45.5)	2.16 (0.69–6.78)	0.188
ADA (> 15 U/L)	1 (5.0)	4 (6.1)	4 (40.0)	7.95 (1.79–35.24)	0.006
γ-GTP (> 45 U/L for females and > 60 U/L for males)	2 (33.3)	5 (12.2)	1 (14.3)	0.43 (0.08–2.46)	0.344
Troponin T (> 0.014 ng/mL)	1 (10.0)	2(4.3)	2 (25.0)	3.24 (0.46–22.65)	0.236
Serum iron(< 7.8umol/L for females and > 10.6 umol/L for males)	3 (30.0)	17 (40.5)	5 (71.4)	2.64 (0.79–8.78)	0.113
Serum creatinine (> 81 umol/L for females and > 111 umol/L for males)	0 (0)	4 (5.6)	1 (9.1)	3.26 (0.48–21.97)	0.225
Urine creatinine (< 2470umol/L for females and < 3450 umol/L for males)	1 (9.1)	3 (5.7)	0 (0)	0.49 (0.05–4.44)	0.524
Reduced haemoglobin (> 7%)	0 (0)	4(5.7)	3(27.3)	8.33 (1.64–42.30)	0.011
Lactate (> 1.6 mmol/L)	4(25.0)	14(20.0)	3(27.3)	1.01 (0.35–2.94)	0.982
IgG (> 16 g/L)	0 (0)	3 (5.4)	1 (12.5)	4.45 (0.53–37.11)	0.168
GPDA (< 44 U/L)	0 (0)	1 (1.5)	0 (0)	1.51 (0.02–105.39)	0.849
FFA (> 769 umol/L)	1 (5.0)	3 (4.5)	0 (0)	0.60 (0.08–4.67)	0.625
β2-microglobulin (> 3 mg/L)	0 (0)	6 (8.5)	1 (9.1)	2.65 (0.51–13.83)	0.248
Uric acid (< 154.7umol/L for females and < 208.3 umol/L for males)	1 (4.8)	7 (9.7)	2 (18.2)	2.39 (0.58–9.89)	0.230
eGFR (< 90 ml/min)	1 (4.8)	16 (22.2)	3 (27.3)	2.66 (0.89–7.96)	0.079
DBIL (> 6.84 umol/L)	5 (23.8)	15 (20.8)	5 (45.5)	1.64 (0.61–4.37)	0.324

*OR* Odds ratio, *CI* Confidence interval, *BMI* Body mass index, *WBC* White blood cell, *CRP* C-reactive protein, *LDH* Lactate dehydrogenase, *SAA* Serum amyloid A, *ALB* Albumin, *ALB/GLB* Albumin/Globulin, *TC* Total cholesterol, *HDL-C* High density lipoprotein cholesterol, *PALB* Prealbumin; *RBP* Retinol binding protein, *Apo A1* Apolipoprotein A1, *GLB* Globulin, *FIB* Fibrinogen, *ALT* Alanine aminotransferase, *AST* Aspartate aminotransferase, SaO_2_ = Oxygen saturation, *PaO*_*2*_ Partial pressure of oxygen in arterial blood, *FiO*_*2*_ Inspired oxygen fraction, *ADA* Adenosine deaminase; *PaCO2* Partial pressure of carbon dioxide, *γ-GTP* γ-glutamyltranspeptidase, *IgG* Immunoglobulin G, GPDA = Glycyl-proline-dipeptidyl aminopeptidase, *FFA* Free fatty acids, *eGFR* Estimated glomerular filtration rate, *DBIL* Direct bilirubin

^a^Data were presented as median (IQR), or n (%) where appropriate

^b^Comorbidity was defined as having at least one of the following diseases: diabetes, hypertension, cardiovascular disease, severe congenital disease, cancer and chronic liver, renal, respiratory disease

### Model construction and evaluation

Based on the criteria described in the Methods, 18 candidate markers and 90 patients were selected for the model construction. Because of similar clinical function, D-dimer and FIB were combined into a new variable of coagulation function as DFIB. Abnormal DFIB was defined as patients with abnormal D-dimer or FIB. Electrolyte disturbance was calculated based on the sum of abnormalities in calcium, phosphorus, potassium, sodium and chlorine. Thus, 16 markers were included in LASSO regression for further feature selection. After 1000 resamples by bootstrap, ALB, CRP, LDH, DFIB, comorbidity, lymphocyte count, eosinophil count, and electrolyte disturbance were finally selected as the predictors in the model. The detailed frequency of each marker in the 1000 LASSO models is summarised in Additional file 1: Tables S2 and S3.

Table 3 presents the performance of each model in the internal and external validations. For the internal validation, high levels of AUROCs were found among four models of logistic regression, ridge regression, support vector machine, and random forest from 0.919 (95% CI 0.793–0.955) to 0.973 (95% CI 0.935–0.993). For the external validation, the ridge regression model showed the best performance with the highest AUROC of 0.827 (95% CI 0.716–0.921). Therefore, the ridge regression model was considered as the best model because of its high predictive power.

**Table 3:** Performance of different models in the internal and external validations

	Logistic regression	Ridge regression	Random forest	Support vector machine
Internal validation				
AUROC (95% CI)	0.919 (0.793–0.955)	0.930 (0.914–0.943)	0.973 (0.935–0.993)	0.955 (0.892–0.993)
External validation				
AUROC (95% CI)	0.756 (0.617–0.869)	0.827 (0.716–0.921)	0.795 (0.669–0.905)	0.802 (0.682–0.908)

*AUROC* Area under the receiver operator characteristic curve, *CI* Confidence interval

A risk score was then calculated according to the result of the ridge regression model using the following formula:

$$\begin{gathered} {\text{Risk score}}\, = \,{26}.{78}\, \times \,{\text{lactate dehydrogenase}}\, + \,{19}.{31}\, \times \,{\text{C}} - {\text{reactive protein}}\, + \,{17}.{16}\, \times \,{\text{DFIB}}\, \hfill \\ + \,{19}.{81}\, \times \,{\text{albumin}}\, + \,{17}.{59}\, \times \,{\text{comorbidity}}\, + \,{9}.{19}\, \times \,{\text{eosinophil count}}\, + \,{4}.{83}\, \times \,{\text{electrolyte}} \hfill \\ {\text{disturbance}}\, + \,{6}.{25}\, \times \,{\text{lymphocyte count}} \hfill \\ \end{gathered}$$

All markers, except electrolyte disturbance, were in dichotomous forms (1 = abnormal, 0 = normal). The range of electrolyte disturbance was from 0 to 5. Figure 2 presents the receiver operating characteristic curve (A) and calibration curve (B) of the risk score. The risk score indicated good discrimination of severe or critical type with an AUROC of 0.897 (95% CI 0.845–0.940). In addition, calibration curve graphically showed good consistency between the predicted and actual probabilities of severe or critical type. Using the optimal cutoff value of 71, the sensitivity of the risk score was 87.1%, and specificity was 78.1% for the COVID-19 severity prediction. Figure 3 presents the distribution of risk scores in different degrees of COVID-19 severity. The mild patients had the lowest median risk score of 9.19 (IQR: 0–26.82), then after the moderate (median: 45.65, IQR: 19.56–76.91) and severe patients (median: 102.38, IQR: 81.37–120.92). The critical patients had the highest median risk score of 113.42 (IQR: 87.89–125.75). In order to help clinicians to detect the patients who were likely to develop severe or critical COVID-19 at admission, we developed a web-based assessment system based on our risk score. (Fig. 4, Website: http://www.gtrsp.com:8011/).

## Discussion

Early identification of patients who were likely to develop severe or critical COVID-19 would help reduce the case-fatality rate and efficiently utilize the limited medical resources. In this study, we identified a panel of clinical markers associated with the severity of COVID-19 and constructed different severity-prediction models. We found that the ridge regression model was the best based on high AUROCs in both the internal and external validations of 0.930 (95% CI, 0.914–0.943) and 0.827 (95% CI, 0.716–0.921), respectively. Furthermore, we established a risk score and a web-based assessment system to help clinicians to detect the patients who were likely to develop severe or critical COVID-19 at admission.

Previous studies showed that severe or critical COVID-19 patients were older, had more comorbidities, higher levels of LDH, D-dimer, CRP, and lower levels of ALB, lymphocyte count [3–6, 8]. These findings were consistent in our study. Moreover, using the data of 208 patients from Fuyang, Anhui Province, Ji et al. [12] established a scoring model named as CALL to predict the severity of COVID-19. Dong et al. [13] also developed a scoring system with the data of 147 patients from Wuhan, Hubei Province. The AUROCs of their models were 0.910 and 0.843, slightly lower than the AUROC of our assessment model in the internal validation (0.930). However, their models were not validated in an external dataset, leading to the limitation of their generalizability. In contrast, our model validated in an independent dataset and obtained a satisfactory AUROC of 0.827.

Among the eight markers in our model, LDH, CRP, ALB, and lymphocyte count were well-recognized predictors for COVID-19 severity [16]. For eosinophil count, Zhu et al. [14] demonstrated that decreased eosinophils could induce acute lung injury in the mouse model. Liu et al. [15] also found that increased eosinophil count predicted the improvement in COVID-19 progression. Several studies reported that severe or critical COVID-19 patients often experienced electrolyte disturbances [3, 4, 16]. In our study, we used the sum of abnormalities in potassium, calcium, sodium, phosphorus and chlorine to comprehensively evaluate the degree of electrolyte disturbances. D-dimer and FIB were indicators of coagulation function. Chen et al. [8] reported that patients infected with SARS-CoV-2 had abnormal coagulation function (hypercoagulation). We combined the two indicators to increase the sensitivity of judging abnormal coagulation and avoid the collinearity of the two markers. Different from other studies, age was not included in our final model. This might be owing to the high correlation between age and comorbidity in the training set, and the LASSO regression identified comorbidity as a more important marker.

There were several limitations in our study. First, there were different distribution on the severity of COVID-19 between the training and validation sets. There were no critical cases in training set while without mild cases in validation set. This difference was due to the rule of government on the COVID-19 prevention and control in Zhejiang Province in China. Xixi Hospital (municipal-level hospital for infectious diseases) mainly receive and cure the patients with mild, moderate, and severe COVID-19 (no critical patients), while FAHZJU (provincial-level hospital) is mainly responsible for moderate, severe, and critical patients (no mild patients). The different distribution of the severity might have influences on the model construction and validation. However, even there were these differences, the ideal performance was still obtained in the validation stage, and this result indicated that there was relatively high generalizability in our model. Second, the subjects were mainly recruited from Hangzhou and the sample size was relatively small. This would limit the generalizability of our model. Additional validation from areas outside Zhejiang should be conducted in the future. Third, because of the retrospective study design, some laboratory tests were not done in some patients. Therefore, their associations with the severity of COVID-19 might be misestimated. Fourth, the clinical data of the subjects were not comprehensive. Adding other specific markers such as cytokines might improve the performance of our model. Finally, due to the low prevalence of comorbidity, the risks in different types of comorbidities were not considered in the assessment model.

## Conclusions

In this study, we screened eight severity-associated clinical markers of lactate dehydrogenase, C-reactive protein, albumin, comorbidity, electrolyte disturbance, coagulation function, eosinophil and lymphocyte counts in COVID-19 patients. Based on these eight markers, an assessment model was constructed to help the clinician to evaluate the likelihood of developing severity of COVID-19 at admission and early take measures on clinical treatment.

## Supplementary Information

**Additional file 1:** **Table S1.** Associations of continuous laboratory markers with the severity of COVID-19 in the training set. **Table S2.** Remaining frequency of the 16 markers in 1000 LASSO regression models. **Table S3.** Associations of the selected eight markers with the severity of COVID-19 in the external validation set.

## Data Availability

The datasets used and/or analyzed during the current study are available from the corresponding authors on reasonable request.

## Abbreviations

ALB: Albumin
AUROC: Area under the receiver operator characteristic curve
BMI: Body mass index
CT: Computerized tomography
CI: Confidence interval
COVID-19: Coronavirus disease 2019
CRP: C-reactive protein
FIB: Fibrinogen
FiO_2_: Inspired oxygen fraction
IQR: Interquartile range
LASSO: Least Absolute Shrinkage and Selection Operator
LDH: Lactate dehydrogenase
OR: Odds ratio
PaO_2_: Partial pressure of oxygen in arterial blood
SaO_2_: Oxygen saturation
SARS-CoV-2: Severe acute respiratory syndrome coronavirus 2
WHO: World Health Organization

## References

[1] Zhu N, Zhang D, Wang W, Li X, Yang B, Song J, Zhao X, Huang B, Shi W, Lu R (2020). A novel coronavirus from patients with pneumonia in China, 2019. N Engl J Med.

[2] World Health Organization: Novel Coronavirus (2019-nCoV). Situation report-133.2020. https://www.who.int/docs/default-source/coronaviruse/situation-reports/20200701-covid-19-sitrep-163.pdf?sfvrsn=9a56f2ac_4. Accessed 2 Jul 2020

[3] Wu ZY, McGoogan JM (2020). Characteristics of and important lessons from the coronavirus disease 2019 (COVID-19) outbreak in China summary of a report of 72 314 cases from the Chinese Center for Disease Control and Prevention. JAMA.

[4] Huang C, Wang Y, Li X, Ren L, Zhao J, Hu Y, Zhang L, Fan G, Xu J, Gu X (2020). Clinical features of patients infected with 2019 novel coronavirus in Wuhan. China Lancet.

[5] Guan WJ, Ni ZY, Hu Y (2020). Clinical characteristics of coronavirus disease 2019 in China. N Engl J Med.

[6] Xu XW, Wu XX, Jiang XG, Xu KJ, Ying LJ, Ma CL, Li SB, Wang HY, Zhang S, Gao HN (2020). Clinical findings in a group of patients infected with the 2019 novel coronavirus (SARS-Cov-2) outside of Wuhan, China: retrospective case series. BMJ.

[7] Wang DW, Hu B, Hu C, Zhu FF, Liu X, Zhang J, Wang BB, Xiang H, Cheng ZS, Xiong Y (2020). Clinical characteristics of 138 hospitalized patients with 2019 novel coronavirus-infected pneumonia in Wuhan. China JAMA.

[8] National Health Commission of the People’s Republic of China: Chinese management guideline for COVID-19 (version 7.0) [in Chinese]. http://www.nhc.gov.cn/yzygj/s7653p/202003/46c9294a7dfe4cef80dc7f5912eb1989/files/ce3e6945832a438eaae415350a8ce964.pdf. Accessed 15 Mar 2020.

[9] Chen NS, Zhou M, Dong X, Qu JM, Gong FY, Han Y, Qiu Y, Wang JL, Liu Y, Wei Y (2020). Epidemiological and clinical characteristics of 99 cases of 2019 novel coronavirus pneumonia in Wuhan, China: a descriptive study. Lancet.

[10] Chen ZH, Li YJ, Wang XJ (2020). Chest CT of COVID-19 in patients with a negative first RT-PCR test: Comparison with patients with a positive first RT-PCR test. Medicine (Baltimore).

[11] Chen Z, Fan H, Cai J (2020). High-resolution computed tomography manifestations of COVID-19 infections in patients of different ages. Eur J Radiol.

[12] Zheng S, Fan J, Yu F (2020). Viral load dynamics and disease severity in patients infected with SARS-CoV-2 in Zhejiang province, China, January-March 2020: retrospective cohort study. BMJ.

[13] World Health Organization: Clinical management of severe acute respiratory infection when novel coronavirus (2019-nCoV) infection is suspected: interim guidance. 2020. https://www.who.int/docs/default-source/coronaviruse/clinical-management-of-novel-cov.pdf. Accessed 15 Mar 2020

[14] Ji D, Zhang D, Xu J (2020). Prediction for progression risk in patients with COVID-19 pneumonia: the CALL score. Clin Infect Dis.

[15] Dong Y, Zhou H, Li M (2020). A novel simple scoring model for predicting severity of patients with SARS-CoV-2 infection. Transbound Emerg Dis.

[16] Zhu C, Weng QY, Zhou LR (2020). Homeostatic and early recruited CD101—eosinophils suppress endotoxin-induced acute lung injury. Eur Respir J.

[17] Sun S, Cai X, Wang H (2020). Abnormalities of peripheral blood system in patients with COVID-19 in Wenzhou. China Clin Chim Acta.

[18] Liu F, Xu A, Zhang Y (2020). Patients of COVID-19 may benefit from sustained Lopinavir-combined regimen and the increase of Eosinophil may predict the outcome of COVID-19 progression. Int J Infect Dis.

[19] Lippi G, South AM, Henry BM (2020). Electrolyte imbalances in patients with severe coronavirus disease 2019 (COVID-19). Ann Clin Biochem.

