# Supplementary material for: Severity-associated markers and assessment model for predicting the severity of COVID-19: a retrospective study in Hangzhou, China
Source: BMC Infect Dis. 2021 Aug 9;21:774. doi: 10.1186/s12879-021-06509-6 (PMC8350279; doi:10.1186/s12879-021-06509-6)
Supplement: Supplementary file 1 — Additional file 1: Table S1. Associations of continuous laboratory markers with the severity of COVID-19 in the training set. Table S2. Remaining frequency of the 16 markers in 1000 LASSO regression models. Table S3. Associations of the selected eight markers with the severity of COVID-19 in the external validation set. [file 12879_2021_6509_MOESM1_ESM.docx]

**Table S1.** Associations of continuous biochemical markers with the severity of COVID-19 in the training set

|  | N | Mild  median (IQR) | Moderate  median (IQR) | Severe  median (IQR) | P_1_ value ^a^ | P_2_ value ^b^ |
| --- | --- | --- | --- | --- | --- | --- |
| **Inflammation** |  |  |  |  |  |  |
| CRP (mg/L) | 104 | 1(1-8) | 9(4-19) | 55(19.5-72) | <0.001 | <0.001 |
| LDH (U/L) | 103 | 137(123-183) | 166(145-205.5) | 283(228-314.5) | <0.001 | <0.001 |
| PCT (ng/mL) | 98 | 0.03(0.02-0.04) | 0.03(0.02-0.04) | 0.07(0.05-0.08) | <0.001 | 0.330 |
| SAA (mg/L) | 104 | 11(5-28) | 58.5(21.25-112) | 111(75-137) | <0.001 | <0.001 |
| Lactate (mmol/L) | 97 | 1.45(1-1.63) | 1.2(0.82-1.5) | 1.3(1.15-1.8) | 0.291 | 0.617 |
| β2-microglobulin (mg/L) | 103 | 1.49(1.34-1.91) | 1.82(1.35-2.38) | 2.33(2.17-2.72) | 0.002 | 0.003 |
| WBC count (10⁹/L) | 104 | 6.26(5.17-8.43) | 5.24(4.44-6.35) | 4.98(4.38-6.04) | 0.057 | 0.027 |
| Eosinophil count (10⁹/L) | 104 | 0.07(0.03-0.16) | 0.01(0-0.03) | 0(0-0.01) | <0.001 | <0.001 |
| Basophil count (10⁹/L) | 104 | 0.02(0.01-0.03) | 0.01(0-0.01) | 0(0-0.01) | <0.001 | <0.001 |
| Monocyte count (10⁹/L) | 104 | 0.47(0.43-0.57) | 0.39(0.31-0.51) | 0.31(0.24-0.5) | 0.059 | 0.244 |
| Neutrophils count (10⁹/L) | 104 | 3.28(2.75-6.66) | 3.42(2.62-4.34) | 3.78(3.46-4.09) | 0.765 | 0.387 |
| Lymphocyte count (10⁹/L) | 104 | 1.64(1.1-2.31) | 1.21(0.84-1.66) | 0.65(0.58-1.22) | 0.002 | 0.001 |
| IgA (g/L) | 82 | 1.98(1.5-2.58) | 2.25(1.83-2.69) | 2.59(2.06-2.75) | 0.338 | 0.166 |
| IgG (g/L) | 82 | 11.2(9.77-11.83) | 12.4(11.11-13.84) | 11.07(10.31-12.88) | 0.079 | 0.145 |
| IgM (g/L) | 82 | 1.26(0.97-1.55) | 1.19(0.9-1.62) | 1.27(1.05-1.64) | 0.938 | 0.809 |
| Complement B (g/L) | 82 | 0.34(0.32-0.38) | 0.37(0.35-0.4) | 0.37(0.37-0.39) | 0.103 | 0.104 |
| Complement C1q (mg/L) | 82 | 247.4(215.72-273.3) | 249.1(220-275.82) | 264.9(208.15-287.57) | 0.871 | 0.733 |
| Complement C3 (g/L) | 82 | 1.13(1-1.32) | 1.15(1.06-1.35) | 1.13(1.09-1.42) | 0.777 | 0.531 |
| Complement C4 (g/L) | 82 | 0.38(0.26-0.4) | 0.38(0.31-0.49) | 0.55(0.49-0.61) | 0.006 | 0.003 |
| **Electrolyte** |  |  |  |  |  |  |
| Calcium (mmol/L) | 100 | 2.4(2.34-2.49) | 2.24(2.18-2.33) | 2.12(2.1-2.15) | <0.001 | <0.001 |
| **Table S1 Continued** |  |  |  |  |  |  |
| Phosphorus (mmol/L) | 100 | 1.19(0.99-1.37) | 0.93(0.84-1.09) | 0.86(0.81-0.98) | <0.001 | <0.001 |
| Sodium (mmol/L) | 104 | 138.2(136.4-139.5) | 137.9(136.6-139.22) | 135.1(132.65-137.2) | 0.009 | 0.001 |
| Chlorine (mmol/L) | 104 | 103.6(102.1-105) | 103.1(101.1-104.2) | 100.9(99.6-102.85) | 0.050 | 0.009 |
| Potassium (mmol/L) | 104 | 3.92(3.58-4.49) | 3.79(3.47-3.96) | 3.58(3.48-3.88) | 0.197 | 0.036 |
| Serum iron (umol/L) | 59 | 16.95(9.92-22.42) | 11.35(6.03-16.7) | 4.7(3.05-7.1) | 0.016 | 0.006 |
| **Nutritional metabolism** |  |  |  |  |  |  |
| ALB (g/L) | 104 | 43.3(42.3-45.8) | 40.4(37.6-43.62) | 35.6(34-36.3) | <0.001 | <0.001 |
| GLB (g/L) | 104 | 25.9(24.9-29.8) | 30.3(27.95-32.45) | 32.4(30.2-34.05) | 0.002 | 0.001 |
| ALB/GLB | 104 | 1.6(1.5-1.8) | 1.3(1.17-1.5) | 1.1(1-1.2) | <0.001 | <0.001 |
| PALB (mg/L) | 97 | 212(180-256.25) | 159.5(140.25-185.5) | 127(88-142.5) | <0.001 | <0.001 |
| RBP (mg/L) | 96 | 28.05(23.62-35.92) | 22.55(19.02-26.35) | 15.5(12.83-19.7) | 0.003 | <0.001 |
| TC (mmol/L) | 104 | 4.42(4.06-4.76) | 4.17(3.57-4.92) | 3.5(3.16-3.92) | 0.033 | 0.031 |
| TG (mmol/L) | 104 | 0.88(0.64-1.61) | 1.08(0.87-1.58) | 1.15(0.79-1.56) | 0.425 | 0.538 |
| FFA (umol/L) | 96 | 260.5(120.5-444.25) | 348(123.75-472.25) | 446.5(280.25-610.5) | 0.209 | 0.222 |
| HDL-C (mmol/L) | 104 | 1.27(1.08-1.55) | 1.12(0.97-1.23) | 0.82(0.68-1) | <0.001 | <0.001 |
| LDL-C (mmol/L) | 104 | 2.24(2.06-2.6) | 2.21(1.88-2.8) | 1.97(1.73-2.45) | 0.393 | 0.281 |
| Apo A1 (g/L) | 96 | 1(0.9-1.08) | 0.9(0.84-1.04) | 0.72(0.62-0.79) | <0.001 | 0.001 |
| Apo B (g/L) | 96 | 0.76(0.7-0.92) | 0.77(0.66-1) | 0.7(0.58-0.89) | 0.464 | 0.401 |
| Apo E (mg/L) | 96 | 48.25(41-56.92) | 45.95(39.58-56.92) | 41.35(33.3-49.23) | 0.312 | 0.230 |
| Lipase (U/L) | 100 | 27.3(23.9-33.05) | 33.85(26.83-41.52) | 35.3(32-60) | 0.043 | 0.420 |
| Amylase (U/L) | 100 | 42(34-61) | 53.5(44.25-65) | 47(36.5-68.5) | 0.223 | 0.514 |
| RBC count (10⁹/L) | 104 | 4.69(4.56-4.91) | 4.53(4.21-4.89) | 4.58(4.44-4.7) | 0.193 | 0.301 |
| HCT (L/L) | 97 | 0.44(0.42-0.45) | 0.4(0.38-0.46) | 0.42(0.4-0.44) | 0.250 | 0.543 |
| RDW-CV (%) | 104 | 12.4(12-12.7) | 12.2(11.8-12.6) | 11.8(11.55-12.3) | 0.149 | 0.540 |
| Hemoglobin (g/L) | 104 | 144(133-147) | 134(125-147) | 138(131-144) | 0.383 | 0.576 |
| **Table S1 Continued** |  |  |  |  |  |  |
| Platelet count (10⁹/L) | 104 | 214(203-242) | 189.5(164-234.25) | 157(125.5-215) | 0.021 | 0.043 |
| PDW (%) | 104 | 13.1(11.5-14.3) | 12.65(11.28-13.85) | 13.2(12.35-14.45) | 0.285 | 0.970 |
| **Liver function** |  |  |  |  |  |  |
| ALT (U/L) | 104 | 18(14-42) | 17.5(11.75-29) | 26(13.5-49) | 0.332 | 0.099 |
| AST (U/L) | 104 | 22(18-32) | 23.5(17-31) | 35(22.5-47) | 0.229 | 0.824 |
| γ-GTP (U/L) | 54 | 32(20-69.5) | 20(13-32) | 20(12-30) | 0.540 | 0.083 |
| GPDA (U/L) | 96 | 87.7(72.35-112.83) | 64.5(57.67-77.72) | 59.3(55.43-67.6) | 0.001 | <0.001 |
| Cholinesterase (U/L) | 96 | 7619.5(7148.25-8908.75) | 7343(6656-8577.5) | 6451(5196-6943.75) | 0.004 | 0.001 |
| ADA (U/L) | 96 | 8.9(8.17-11.87) | 9.65(8.27-11.07) | 11.6(8.7-16.1) | 0.222 | 0.026 |
| ALP (U/L) | 104 | 76(66-180) | 72(56.75-84.25) | 74(65-93) | 0.114 | 0.002 |
| α-L- fucosidase (U/L) | 96 | 19.9(17.7-24.75) | 21.8(17.7-26.2) | 21.9(20.32-26.65) | 0.611 | 0.384 |
| Total bile acids (umol/L) | 96 | 3.2(2.18-4.95) | 3.25(2-4.85) | 3.65(2.3-6.75) | 0.743 | 0.676 |
| TBIL (umol/L) | 104 | 9.29(6.01-19.5) | 11.88(7.34-17.52) | 11.73(7.21-18.52) | 0.889 | 0.710 |
| DBIL (umol/L) | 104 | 3.26(2.4-6.69) | 4.75(2.94-6.37) | 5.84(3.43-7.53) | 0.386 | 0.229 |
| IBIL (umol/L) | 104 | 6.1(3.5-12.1) | 6.85(4.3-10.77) | 5.9(3.8-11) | 0.932 | 0.929 |
| **Renal function** |  |  |  |  |  |  |
| Serum urea (mmol/L) | 104 | 4(3.1-4.9) | 3.9(3.08-4.7) | 4.3(3.55-6.85) | 0.436 | 0.068 |
| Serum creatinine | 104 | 61(44-77) | 61(51.75-78) | 68(58.5-75.5) | 0.388 | 0.178 |
| Urine creatinine | 68 | 13544(10711-15583) | 8402(4242-14588) | 5124(4598.25-5785.25) | 0.156 | 0.032 |
| eGFR (ml/min) | 104 | 124(103-184) | 107.5(95.25-122) | 106(94-117) | 0.023 | 0.002 |
| Uric acid (umol/L) | 104 | 299.1(273.7-343.9) | 250.3(212.2-322.52) | 240.6(187-263.15) | 0.049 | 0.016 |
| **Cardiac function** |  |  |  |  |  |  |
| Troponin T (ng/mL) | 64 | 0(0-0.01) | 0(0-0.01) | 0.01(0.01-0.01) | 0.157 | 0.419 |
| Troponin I (ng/mL) | 74 | 0.01(0.01-0.01) | 0.01(0.01-0.01) | 0.01(0.01-0.01) | 0.097 | 0.476 |
| Creatine kinase (U/L) | 104 | 72(54-82) | 65(48.75-89.5) | 86(57-89.5) | 0.445 | 0.528 |
| **Table S1 Continued** |  |  |  |  |  |  |
| Creatine kinase-MB (U/L) | 103 | 11(9-14) | 12(9-15) | 14(11-18) | 0.416 | 0.308 |
| **Respiratory function** |  |  |  |  |  |  |
| SaO_2_ (%) | 97 | 98.75(97.33-99.28) | 98.3(97.12-98.97) | 97(94.15-97.95) | 0.018 | 0.116 |
| PaO_2_/FiO_2_ (mmHg) | 97 | 557.14(456.31-748.03) | 453.33(375.11-578.57) | 390.95(297.14-466.19) | 0.002 | 0.003 |
| PaCO_2_ (mmHg) | 97 | 42.25(40.2-43.3) | 39.75(37.32-42.25) | 35.8(34.4-37.1) | 0.000 | 0.004 |
| Reduced hemoglobin (%) | 97 | 1.25(0.73-2.68) | 1.7(1.02-2.88) | 3(2.05-5.85) | 0.018 | 0.113 |
| COHb (%) | 97 | 0.55(0.3-0.75) | 0.6(0.5-0.8) | 0.7(0.5-0.8) | 0.628 | 0.603 |
| **Coagulation function** |  |  |  |  |  |  |
| TT (s) | 104 | 18.2(17.6-18.6) | 17.4(17.1-17.92) | 16.6(16.5-17.35) | 0.002 | 0.001 |
| PT (s) | 104 | 11.8(11.5-12.4) | 11.8(11.5-12.5) | 12.6(11.65-12.95) | 0.245 | 0.107 |
| APTT (s) | 104 | 29.7(28.6-31) | 30.1(27.75-32.2) | 33.1(30.45-35.5) | 0.017 | 0.007 |
| FIB (g/L) | 104 | 2.03(1.87-2.73) | 3.15(2.48-4.07) | 4.37(3.64-5.04) | <0.001 | <0.001 |
| D-dimer (mg/L) | 104 | 0.14(0.1-0.18) | 0.24(0.15-0.42) | 0.58(0.36-0.8) | <0.001 | 0.020 |
| INR | 104 | 1(0.97-1.05) | 1(0.97-1.06) | 1.07(0.98-1.1) | 0.251 | 0.094 |

^a^ P_1_ value was calculated by Kruskal-Wallis test.

^b^ P_2_ value was calculated by ordinal logistic regression model.

CRP = C-reactive protein; LDH = lactate dehydrogenase; PCT = procalcitonin; SAA = serum amyloid A; WBC = white blood cell; IgA = immunoglobulin A; IgG = immunoglobulin G; IgM = immunoglobulin M; ALB = albumin; GLB = globulin; PALB = prealbumin; RBP = retinol binding protein; TC = total cholesterol; TG = triglyceride; FFA = free fatty acids; HDL-C = high density lipoprotein cholesterol; LDL-C = low density lipoprotein cholesterol; Apo A1 = apolipoprotein A1; Apo B = apolipoprotein B; Apo E = apolipoprotein E; RBC = red blood cell; HCT = Hematocrit; RDW-CV = red blood cell distribution width coefficient variation; PDW = platelet distribution width; ALT = Alanine aminotransferase; AST = aspartate aminotransferase; γ-GTP = γ-glutamyltranspeptidase; GPDA = Glycyl-proline-dipeptidyl aminopeptidase; ADA = adenosine deaminase; ALP = alkaline phosphatase; TBIL = total bilirubin; DBIL = direct bilirubin; IBIL = indirect bilirubin; eGFR = estimated glomerular filtration rate; SaO2 = oxygen saturation; PaO2 = partial pressure of oxygen in arterial blood; FiO2 = inspired oxygen fraction; PaCO2 = partial pressure of carbon dioxide; COHb = carboxyhemoglobin; TT = Thrombin time; PT = Prothrombin time; APTT = activated partial thromboplastin time; FIB = fibrinogen; INR = international normalized ratio

**Table S2.** Remaining frequency of the 16 markers in 1,000 LASSO regression models

|  | Remaining frequency ^a^ |
| --- | --- |
| Albumin | 0.947 |
| C-reactive protein | 0.835 |
| Lactate dehydrogenase | 0.811 |
| DFIB ^b^ | 0.778 |
| Comorbidity ^c^ | 0.581 |
| Lymphocyte count | 0.525 |
| Eosinophil count | 0.523 |
| Electrolyte disturbances | 0.509 |
| Retinol conjugated protein | 0.469 |
| High density lipoprotein cholesterol | 0.433 |
| Albumin/Globulin | 0.406 |
| Age | 0.374 |
| Apolipoprotein A1 | 0.348 |
| Fever | 0.239 |
| Prealbumin | 0.103 |
| Serum amyloid A | 0 |

^a^ Remaining frequency was the frequency of the marker that were present in 1000 bootstraps

^b^ DFIB was the combined marker of d-dimer and fibrinogen.

^c^ Comorbidity was defined as having at least one of the following disease: diabetes, hypertension, cardiovascular disease, severe congenital disease, cancer and chronic liver, renal, respiratory disease.

**Table S3.** Associations of the selected eight markers with the severity of COVID-19 in the external validation set

|  | Moderate | Severe or critical | OR (95% CI) | P value |
| --- | --- | --- | --- | --- |
| Number | 16 | 52 |  |  |
| ALB (<40 g/L), n(%) | 6(37.5) | 33(63.5) | 2.90(0.91-9.22) | 0.072 |
| CRP (>10 mg/L) , n(%) | 11(68.8) | 49(94.2) | 7.42(1.54-35.82) | 0.013 |
| LDH (>250 U/L) , n(%) | 4(25.0) | 37(71.2) | 7.40(2.06-26.64) | 0.002 |
| Abnormal DFIB ^a^, n(%) | 10(62.5) | 45(86.5) | 3.86(1.06-13.98) | 0.040 |
| Comorbidity ^b^ , n (%) | 11(68.8) | 45(86.5) | 2.92(0.78-10.98) | 0.112 |
| Lymphocyte count (<1.1×10⁹/L) , n(%) | 4(25.0) | 39(75.0) | 9.00(2.47-32.83) | 0.001 |
| Eosinophil count (<0.02×10⁹/L) , n(%) | 12(75.0) | 50(96.2) | 8.33(1.36-50.95) | 0.022 |
| Electrolyte disturbance ^c^, median(IQR) | 0.5(0.0-1.0) | 1.0(1.0-2.5) | 2.61(1.27-5.37) | 0.009 |

^a^ DFIB was the combined marker of d-dimer and fibrinogen. Abnormal DFIB was defined as patients with abnormal d-dimer or fibrinogen.

^b^ Comorbidity was defined as having at least one of the following diseases: diabetes, hypertension, cardiovascular disease, severe congenital disease, cancer and chronic liver, renal, respiratory disease.

^c^ Electrolyte disturbance was calculated as the sum of abnormalities in calcium, phosphorus, potassium, sodium and chlorine

OR = Odd ratio; CI = Confidence interval; ALB = Albumin; CRP = C-reactive protein; LDH = Lactate dehydrogenase
